# Vertical migration of some herbicides through undisturbed and homogenized soil columns

**DOI:** 10.2478/v10102-010-0041-z

**Published:** 2010-11

**Authors:** Md. Wasim Aktar, Dwaipayan Sengupta, Swarnali Purkait, Ashim Chowdhury

**Affiliations:** 1 Pesticide Residue Laboratory, Department of Agricultural Chemicals, Bidhan Chandra Krishi Viswavidyalaya, Mohanpur-741252, Nadia, West Bengal, India; 2 Department of Agricultural Chemistry and Soil Science, Institute of Agricultural Science, University of Calcutta, Kolkata, West Bengal, India

**Keywords:** leachability, soil columns, thiocarbamate, dinitroaniline, herbicides, GLC and HPLC analysis

## Abstract

A laboratory experiment was conducted by using three herbicides, two from dinitroaniline group and one from thiocarbamate group to know their degree of downward movement (leachability) through soil columns and their contribution in ground water contamination. Soil columns were loaded with Pendimethalin, Benthiocarb and Oryzalin at doses of 10.0, 10.0 and 7.7 kg/ha, respectively. After 30 days soil samples were analyzed from each segments (i.e. 0–6, 6–12, 12–18, 18–24 and 24–30 cm) for Benthiocarb and Pendimethalin by GLC equipped with Ni^63^ electron capture detector (ECD) and for Oryzalin by HPLC coupled with UV-VIS detector. The results obtained in the present study reveal that the residues of the three herbicides under investigation were predominantly confined to the upper soil layer (0–6 cm). Comparatively, low mobility of these herbicides in soils could be due to strong adsorption of these chemical to soil colloids.

**Abbreviations**
OC: Organic Carbon
CEC: Cation Exchange Capacity
GLC: Gas Liquid Chromatograph
GC: Gas Chromatograph
GC-MS: Gas Chromatography-Mass Spectrometry
HPLC: High Performance Liquid Chromatograph
LC-MS: Liquid Chromatography-Mass Spectrometry
ES: Electro Spray

## Introduction

**Figure 1:**
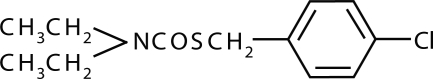
Molecular structure of Benthiocarb.

**Figure 2:**
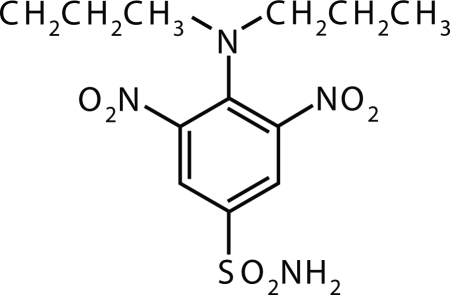
Molecular structure of Oryzalin.

**Figure 3:**
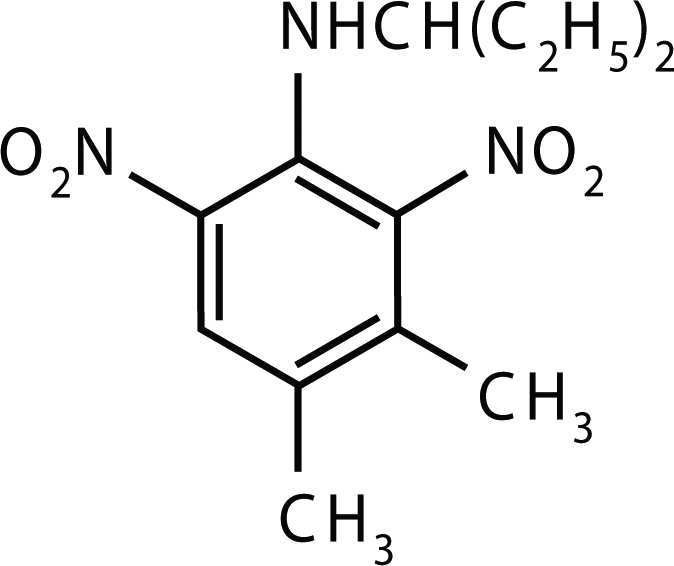
Molecular structure of of Pendimethalin.

The use of pesticides in crop production is inevitable to avoid the losses due to various pests infesting it at different stages of the growth. The extent to which pesticides are susceptible to transport through and from soil, and contribute to nonpoint source pollution, is dependant on the processes of biodegradation and sorption which determine the longevity and mobility of the pesticide in the soil, respectively. The movement of pesticides through soil is an important process that determines their fate in both soil and aquatic environments. Pendimethalin [N-(1-ethylpropyl)-2,6-dinitro-3,4-xylidine], Benthiocarb [3,5-dinitro-N-4,N-4-dipropyl sulfanilamide] and Oryzalin [S-4-chlorobenzyl diethyl thiocarbamate] (Figures 1–3) are used as selective pre-emergence for the control of annual grasses and small seeded broad leaf weeds in cotton, soybean and peanuts (Helling, 1976; Landry *et al*., 2004). This could result in built up of their residues in soil and ground water (Nicholas & Miliadis, 1998; Louchart *et al*., 2004). The purposes of these experiments were to evaluate the downward movement of these herbicides in soil columns thereby predicting the risk of ground water contamination. Leaching studies were conducted in laboratory controlled experiments with undisturbed soil cores.

## Materials and methods

### Design of experiment

Soil sample collected at 0–15 cm depth from farmer's plot at Maricha village, Nadia, West Bengal, India were used after air drying and sieving through 2 mm sieve. A 36 cm long methacrylate column with an inner diameter of 6 cm were cut into six sections (6 cm long each) and sealed with a water-proof silicone adhesive tape. The bottom ring was filled with glass wool. A muslin cloth was fixed with rubber ring on the outer side of columns to avoid contamination of leachate with soil particles. Five rings were hand-packed with sandy loam soil (pH 7.2) collected from 0–15 cm depth after air drying and sieving through 2 mm sieve.

### Application of chemical

Before applying the herbicides, soil columns were wetted to their apparent water holding capacity by applying 0.01 M calcium chloride to the top of the column. After this initial equilibrium, columns were loaded with Pendimethalin, Benthiocarb and Oryzalin at the doses of 10.0, 10.0 and 7.7 kg/ha, respectively. After 24 h of application, the columns were irrigated daily using 0.01 M calcium chloride solution. After 30 days soil samples were analyzed from each segment. The soil of the experimental site was silty clay loam in texture, tending towards neutral in reaction with pH 7.25, OC 0.42% and poses relatively low N status and high P, K status. The soil consists 12% sand, 65% silt and 23% clay having CEC at 25 cmol (p^+^)/µg.

### Extraction and clean-up

A representative sub-soil of 20 g mixed with 0.5 g each of activated charcoal and florisil was filled in a 30 cm long glass column having 1.8 cm i.d. The residues were eluted with 150 mL of n-hexane: acetone (1:1). Leachates collected daily were analyzed by partitioning the residues into dichloromethane phase thrice (100+50+50 mL).The combined organic phase was evaporated to near dryness under reduced pressure at 40 °C (Gustafson, 1989).

### Estimation of residues

**Figure 4:**
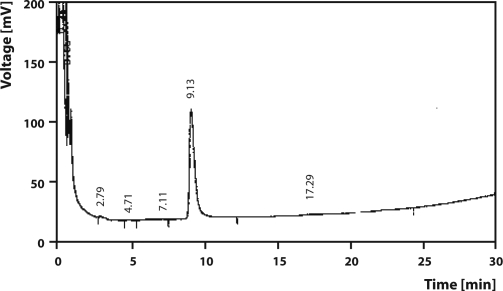
A GLC chromatogram of analytical standard of Benthiocarb.

**Figure 5:**
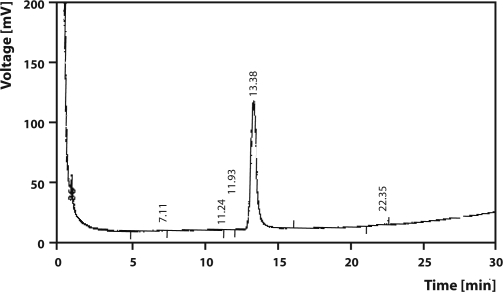
A GLC chromatogram of analytical standard of Pendimethalin.

Final volume was made up by n-hexane and residues were estimated by GLC (Agilent Technologies 6890N Network GC system) with Ni^63^ electron capture detector (ECD) coupled with Chemito 5000 data processor for Benthiocarb and Pendimethalin. The HP-5 capillary column (30 m × 0.32 mm i.d.) of 0.25 µm film thickness was used. The temperatures were: oven 210 °C, injector 225 °C and detector 350 °C. Flow rate of carrier gas (nitrogen) was 1.8 mL/min (split). The retention time, limit of detection (LOD) and limit of quantification (LOQ) were 9.13 min, 0.01 µg/g and 0.04 µg/g for Benthiocarb (Figure 4) and 13.38 min, 0.02 µg/g and 0.07 µg/g for Pendimethalin (Figure 5). The analysis of these herbicides along with their metabolites also carried out by GC-MS (Saturn 2200 model, Varian) for further confirmation. Analysis was carried out as SIS mode with EI Auto ionization mode using Ion Trap as mass detector. The *m/z* values for SIS mode of Analysis Pendimethalin was 162, 191, 252, 253, 282, where for Benthiocarb it was 72, 100, 125, and 257.

**Figure 6:**
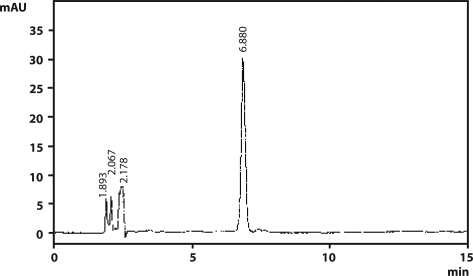
A HPLC chromatogram of analytical standard of Oryzalin.

For analysis of Oryzalin final volume was made up by HPLC grade acetonitrile. Oryzalin residues were estimated using HPLC (model JASCO PU 1580 HPLC pump) with model JASCO UV 1575 UV-VIS detector equipped with Chemito 5000 data processor applying the analytical conditions: column (Shandon Hypersil 250 × 4.6 mm ODS 5, RPC_18_), mobile phase (acetonitrile:water, 7:3), wave length (285 nm) and flow rate at 1 mL/min. The retention time, limit of detection (LOD) and limit of quantification (LOQ) were 6.880 min, 0.01 µg/g and 0.05 µg/g for Oryzalin (Figure 6). The analysis of the herbicide along with their metabolites also carried out by LC-MS (Tandem mass spectrometry, Waters) for further confirmation. Analysis was carried out as SIR mode with ES+ mode using Tandem Mass Detector. The *m/z* values used for SIR mode of Analysis for Oryzalin was 258, 275, 301, 317 and 318.

### Method validation

To work out the extraction efficiency of methods employed for Pendimethalin, Benthiocarb and Oryzalin from soil and leachate, respective sample matrices were spiked in triplicate at two different levels (i.e. at 0.5 and 1.0 µg/mL) with the above mentioned herbicides. The average recoveries of Pendimethalin were above 89 and 94 percent in soil and leachates, respectively. The corresponding recovery values for Benthiocarb and Oryzalin were above 87, 93 and 91, 96%, respectively (Table 1).

**Table 1:** Results of method validation by recovery analysis of three herbicides from various substrates.

Herbicides	Substrates	Amount fortified (in ppm)*	Amount recovered (in ppm)				Recovery of herbicides (Average %)

			R_1_	R_2_	R_3_	Mean ± S.D.	
Pendimethalin	Soil	T_1_ (0.5 ppm)	0.41	0.44	0.45	0.43±0.02	89
		T_2_ (1.0 ppm)	0.86	0.93	0.95	0.91±0.05	
	Leachates	T_1_ (0.5 ppm)	0.45	0.46	0.48	0.46±0.02	94
		T_2_ (1.0 ppm)	0.96	0.94	0.97	0.96±0.02	

Oryzalin	Soil	T_1_ (0.5 ppm)	0.45	0.44	0.46	0.45±0.01	91
		T_2_ (1.0 ppm)	0.88	0.93	0.95	0.92±0.03	
	Leachates	T_1_ (0.5 ppm)	0.48	0.46	0.48	0.47±0.01	96
		T_2_ (1.0 ppm)	0.99	0.96	0.97	0.97±0.02	

Benthiocarb	Soil	T_1_ (0.5 ppm)	0.38	0.43	0.45	0.42±0.03	87
		T_2_ (1.0 ppm)	0.81	0.92	0.96	0.90±0.08	
	Leachates	T_1_ (0.5 ppm)	0.45	0.45	0.48	0.46±0.02	93
		T_2_ (1.0 ppm)	0.91	0.95	0.97	0.94±0.03	

* Amount fortified in μg/g of soil/leachates

### Calculation of residues

The residue content was calculated by using the formula:

$$\text{Residue in ppm (μg/g)= }\frac{{\text{A}}_{\text{1}}\text{ ×C× }{\text{V}}_{\text{1}}}{{\text{A}}_{\text{2}}\text{ ×W× }{\text{V}}_{\text{2}}}\text{×}{\text{R}}_{\text{f}}$$

Where,

*A_1_ = Area of Oryzalin from sample, in chromatogram*

*A_2_ = Area of Oryzalin from standard, in chromatogram*

*V_1_ = Total volume of sample (in mL)*

*C = Concentration of analytical standard in ppm (µg/mL) × µl injected*

*W = Weight of the sample (in gm)*

*V_2_ = Injected volume of sample (in µl)*

*R_f_ = Recovery factor*

Linearity was evaluated by linear regression analysis.

The residue data were subjected to regression analysis and the fit of the data to first order kinetics (Ct = C_o_e^−KI^) was confirmed by testing the statistical significance of correlation coefficient. The half-life values were calculated from dissipation constant calculated from regression analysis.

## Results and discussion

In order to study the downward movement of Pendimethalin, Benthiocarb and Oryzalin in soil columns, residues of the herbicides were determined from 0–6, 6–12, 12–18, 18–24 and 24–30 cm soil column depths after 30 days of the experimentation.

The data presented in Table 2 show that herbicides were mainly confined to the top layer (0–6 cm). Confinement of pesticides in upper plough layer soil was also found in earlier studies (Kogan *et al*., 2007; Landry *et al*., 2006). Pendimethalin and Benthiocarb showed movement up to 18 and 12 cm depth, respectively while Oryzalin penetrated up to 24–30 cm soil depth. Benthiocarb binds to soil organic matter and it is not readily leached into water.

**Table 2:** Depth wise distribution of herbicide residue percentage in soil columns (30 days after application).

Depth (cm)	Residues (%)*		

	Pendimethalin	Benthiocarb	Oryzalin
0–6	97.4	94.2	74.6
6–12	2.5	5.8	21.0
12–18	0.13	BDL	3.9
18–24	BDL	BDL	0.42
24–30	BDL	BDL	BDL

* average of three replicates

BDL= Below Detectable Level

In percentage terms, residues were 97.4 and 94.2 at 0–6 cm depth for Pendimethalin and Benthiocarb respectively. The respective movements for Pendimethalin and Benthiocarb were 2.5 and 5.8 percent at 6–12 cm depth, while at 12–18 cm depth Pendimethalin and Oryzalin showed penetration. In comparison to dinitroaniline group of herbicides, the residues of Oryzalin were 74.6, 21.0 and 3.9 percent, at 0–6, 6–12 and 12–18 cm depths, respectively indicating higher movement.

**Figure 7:**
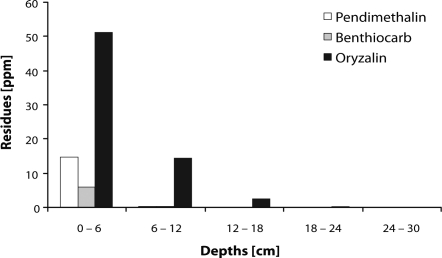
Depth wise distribution of herbicide residues at 30 days after application in soil columns.

The results revealed (Figure 7) that most of the herbicide residues were retained in the upper soil layer probably due to the light irrigation and low water solubility (Pendimethalin 0.3, Benthiocarb 7 and Oryzalin 30 mg/mL) of selected herbicides. The downward movement of Oryzalin in the lowest layer could be due to its polar nature. However, in case of Pendimethalin and Benthiocarb absence of their residues in the lower layers could be attributed to the strong adsorptive nature of dinitroaniline group of herbicides on soil colloids. While working out the recoveries of applied herbicides after 30 days, 29.1, 11.8 and 84.6 percent of Pendimethalin, Benthiocarb and Oryzalin were recovered.

The variation in recoveries among three herbicides could be due to volatile nature of dinitroaniline group, which might have been lost due to volatilization or due to strong adsorption on clay and organic colloids than microbial degradation.

Similar studies carried out by various workers showed >80, >90, 59 and 79 percent residues in 0–5 cm soil layer (Kulshrestha & Yadyraj, 1987; Lu *et al*., 1991; Devendra *et al*., 1997; Devi *et al*., 1997). It was also observed little movement of Pendimethalin in a study carried out on soil column (Hafner, 1990). Oryzalin moved up to 3 cm depth when applied on sandy soil and soils containing high organic matter, while in clayey soil the chemical moved up to 6 cm depth (Fischer, 1972).

The leachates collected up to one month of the experimentation did not reveal residues of any of the herbicides under study. Absence of the herbicide residues in leachates collected beyond 30 cm depth of the soil columns in spite of the high dose of application revealed no risk of groundwater contamination. However, absence of the metabolites of these herbicides in leachates were confirmed by GC-MS and LC-MS.

## Conclusion

The results obtained in the present study reveal that the residues of the three herbicides under investigation were predominantly confined to the upper soil layer (0–6 cm). Comparatively, low mobility of these herbicides in soils could be due to strong adsorption of these chemical to soil colloids. The mobility of Oryzalin was greater in the soil column than Pendimethalin and Benthiocarb due to different soil physio-chemical properties. Comparatively low mobility of these herbicides in soil could be attributed to its strong adsorption by an alluvial soil. Due to low mobility, the chance of leaching of benthiocarb is less than other two herbicides. Therefore, the power of ground water contamination is negligible for Benthiocarb than dinitroaniline group of herbicide.
